# Hip Pro III with animations

**DOI:** 10.1093/jhps/hnv006

**Published:** 2015-02-06

**Authors:** Ian M. Al’Khafaji, Allston J. Stubbs

**Affiliations:** Wake Forest University School of Medicine - Orthopaedic Surgery, Medical Center Boulevard, Winston-Salem, NC, USA

Hip Pro III with animations: application review

Application last updated: 6 November 2014

Developer: SD4Medical in collaboration with Stanford University School of Medicine

Size: 459 MB

Language: English

Compatibility: OS X 10.8 or later; 64-Bit processor

## 

Hip Pro III is a medical application that takes an in-depth look at the anatomy of the hip joint and surrounding structures. This anatomic perspective includes nerves, bones, ligaments, muscles, tendons and blood vessels. It utilizes three-dimensional (3D) images of the associated anatomy to provide the user a 360-degree view. On the side of the screen is a tool bar that contains numerous features that can be used in the program.

When one first enters the main screen, you will see a 3D rendering of the left hip of the human body. The user can apply his finger/cursor to move the image axially 360 degrees. There is a navigation tool that automatically shows an anterior, lateral and posterior view of the hip. A subcategory under this tab is the map feature selection to view each layer independently. There is a cutting tool that allows one to see three different axial cuts, one sagittal cut and one coronal cut of the body. During the entire survey, one can easily apply zoom to view the anatomy more clearly. One limitation of this cross-sectional feature is it only provides five cuts or fields of view of the anatomy that makes following paths of non-orthogonal structures challenging. In regards to image quality, the graphic design and animations are very clear and it is easy to identify different anatomical structures.

One of the exciting features is an add/subtract layer, which allows the user to remove layers of anatomy. As an example, when removing the second layer or ‘L2,’ which contains the gluteus maximus muscle, one can obtain an exceptional view of the external rotators of the hip and the path of the sciatic nerve. While this is a very good feature, one has little freedom to remove individual structures and must rely on the preprogrammed anatomic groups. No matter what layer of anatomy the user is viewing, one can always use the cross-sectional feature to look at the associated anatomy in the field of view. On the very last layer is a view of the osseous anatomy with coloured areas of muscular origins and insertions. This perspective is very useful in medical education and self-studying. In total, there are 105 images of different anatomical structures and a ‘mix’ feature that will overshadow different layers with the first layer as a virtual hip model.

The vessel tool allows viewing of the vascular anatomy of the hip. The vessels do not show up automatically once the layering options are chosen. Layer 5 ‘L5’, which views the lumbosacral plexus and peripheral nerves, is the last layer in which the vessels can be viewed. It would be more useful if the feature allowed the user to view vascular anatomy without other soft tissues obstructing the course and branching of vessels. It would also be helpful if a similar function was programmed for the lumbosacral plexus and peripheral nerves.

The pin function places markers on all structures that are in view. These pins can be selected and shows information such as origin, insertion and mechanism of action. There is a field that allows users to place personal notes and view public notes made by other users. Some pins have a media tab with video of the mechanism of action of selected muscular structures. The media function also shows specific pictures of origins and insertions. These combined functions are the best teaching aspect of the entire application as it provides the needed information of the anatomy in a clear informative manner that is easy to navigate. Further, there is an index function in the tool bar that has every structure listed and will directly go to the pin on the image once it is selected.

One of the least useful tools is the video function. When selected in the tool bar, there is a list of musculoskeletal disease processes and procedures. This list will link to a video which is shown on the main left hip image. Although the animation quality of the video is very clear, many of the videos do not have text or sound describing what is occurring. Thus, the video offering adds more potential confusion than clarity for the user.

Finally, there is a quiz tab found in the tool bar. This allows the user to take multiple choice test questions or dragging answers to pins found on the corresponding anatomy. This automated test is a great function in assessing one’s understanding of hemipelvic anatomy.

Overall, Hip Pro III is very well organized, easy to navigate and provides clear illustrations and animations. The information provided can be a very useful tool for patient and medical professional education specific to basic anatomy of the hip joint. However, the clinical value of the application as an atlas to procedures and disease processes is limited and will benefit from future iterations that provide advanced flexibility of anatomic layering and more comprehensive video functionality.

